# Investigating Methods for Cognitive Workload Estimation for Assistive Robots

**DOI:** 10.3390/s22186834

**Published:** 2022-09-09

**Authors:** Ayca Aygun, Thuan Nguyen, Zachary Haga, Shuchin Aeron, Matthias Scheutz

**Affiliations:** 1 Department of Computer Science, Tufts University, Medford, MA 02155, USA; 2 Department of Electrical and Computer Engineering, Tufts University, Medford, MA 02155, USA

**Keywords:** assistive robots, cognitive workload classification, pupillometry, eye gaze, EEG, multi-modality learning, autonomous interactive systems

## Abstract

Robots interacting with humans in assistive contexts have to be sensitive to human cognitive states to be able to provide help when it is needed and not overburden the human when the human is busy. Yet, it is currently still unclear which sensing modality might allow robots to derive the best evidence of human workload. In this work, we analyzed and modeled data from a multi-modal simulated driving study specifically designed to evaluate different levels of cognitive workload induced by various secondary tasks such as dialogue interactions and braking events in addition to the primary driving task. Specifically, we performed statistical analyses of various physiological signals including eye gaze, electroencephalography, and arterial blood pressure from the healthy volunteers and utilized several machine learning methodologies including *k*-nearest neighbor, naive Bayes, random forest, support-vector machines, and neural network-based models to infer human cognitive workload levels. Our analyses provide evidence for eye gaze being the best physiological indicator of human cognitive workload, even when multiple signals are combined. Specifically, the highest accuracy (in %) of binary workload classification based on eye gaze signals is 80.45 ∓ 3.15 achieved by using support-vector machines, while the highest accuracy combining eye gaze and electroencephalography is only 77.08 ∓ 3.22 achieved by a neural network-based model. Our findings are important for future efforts of real-time workload estimation in the multimodal human-robot interactive systems given that eye gaze is easy to collect and process and less susceptible to noise artifacts compared to other physiological signal modalities.

## 1. Introduction

Assistive robots are envisioned to be deployed in many societal contexts, ranging from co-robots in warehouses and factories to socially assistive robots in health and eldercare settings. While the robots’ tasks will differ depending on the applications, what all of these contexts have in common is the need for the robot to understand the human interactants’ overall cognitive state in order to be able to provide effective help when it is needed. A person who is already busy with an important task and thus potentially overloaded, for example, should not be further stressed by having to answer questions from a robot or having to intervene in a robot’s task, especially if the robot’s task is not all that urgent. Higher cognitive workloads lead to worse human performance [1], and robots causing decreased human performance could quickly be rejected by humans. Thus, understanding when to best communicate or act so as to not overburden the human is a critical component of effective human-robot interaction.

However, there are several challenges involved in assessing human workload. For one, subjective measures such as the NASA-TLX that require humans to answer questions are typically not applicable (as they would exacerbate workload, although they could still be useful after an interaction for an artificial agent to learn about what contexts typically cause high workload). Objective measures utilizing physiological sensors and signal modalities (e.g., [2,3,4,5,6,7]), on the other hand, are not applicable in contexts where humans cannot be instrumented with sensors or they might not yield useful results (e.g., when physiological signals are subjected to significant noise due to motion artifacts). Moreover, even when recording conditions are ideal, it is still unclear which sensing modality and machine learning model to use and whether combining multiple modalities will provide better workload estimates compared to single modalities.

The goal of this paper was to evaluate different machine learning models using multiple physiological signals to determine the combination that yields the best workload estimates. For this purpose, we utilized eye gaze, EEG, arterial blood pressure (heart rate variability (HRV) and blood pressure variability (BPV)) data and their combinations from a multi-tasking simulated driving experiment ([8]; see also our work [9] for some initial analyses that are included here as well). In this experiment, three levels of cognitive workload were experimentally generated by requiring the participants (drivers) to perform secondary tasks such as braking and dialogue interactions at the same time as the primary driving task which induces higher workload levels (that have been verified by delayed reactions to secondary tasks). In addition to performing standard statistical analyses including ANOVAs, Tukey’s honestly significant difference (HSD) and Benjamini–Hochberg multiple pairwise tests, we employed five state-of-the-art machine learning models: (a) *k*-nearest neighbor (*k*-NN), (b) naive Bayes (NB), (c) random forest (RF), (d) support-vector machines (SVM), and (e) neural network-based models (NNMs) to evaluate their ability to detect the different workload levels. Our numerical results together with statistical analysis indicated that eye gaze, particularly pupil diameter, is the most reliable physiological indicator among all considered physiological parameters in assessing human cognitive workload. Moreover, we found out that fusing multiple physiological signals does not enhance the workload classification performance over just using the eye gaze signal alone.

We thus made the following contributions in this paper:

1. We analyzed data from a multi-modal, multi-task controlled driving simulation environment that involves multimodal interaction of several physiological signal modalities including eye gaze (pupillometry), EEG, and arterial blood pressure.
2. Our numerical results and statistical analysis demonstrated the effectiveness of pupil diameter in assessing cognitive workload. Particularly, our empirical results indicated that pupil diameter outperforms other physiological signals in cognitive workload prediction tasks regardless of the learning models.
3. Additionally, we showed that combining several physiological signal modalities does not provide a substantial increase in workload classification performance compared to using just eye gaze alone. This suggested that eye gaze is a sufficient modality for assessing cognitive workload in interactive, multi-modal, multi-task settings.

The remainder of this paper is organized as follows. Section 2 provides a summary of recent works on workload estimation/classification based on various types of physiological signals. Section 3 describes the details of how the driving simulation scenario is designed and how the signals are collected and processed. The workload evaluation, the details of physiological signal pre-processing steps, and feature extraction are summarized in Section 4. Five state-of-the-art machine learning models and their hyper-parameters are described in Section 5. Finally, we provide the numerical result in Section 6 and conclude in Section 7.

## 2. Background and Related Work

*Cognitive workload* is defined as the load imposed on working memory during a cognitive process and is one of the most important factors in designing and implementing robust human-machine systems [10,11]. Assessment of cognitive workload is mainly based on two methods: (a) subjective measurements and (b) objective measurements [2,12]. Subjective measurements use the perceived feeling and self-rating of operators to determine the cognitive workload. For subjective measurements, it is common to predict the cognitive workload by leveraging questions such as the proposed methods in the Administration-Task Load Index [13], Subjective Workload Assessment Technique [14], and Workload Profile [15]. On the other hand, objective measurements are mainly based on the recordings of task performance and physiological signals, such as EEG, heart rate, and eye movement [16,17,18], which we will review below.

### 2.1. EEG

EEG is considered a common and reliable indicator for assessing the human cognitive workload [2,3,4,19]. Berka et al. [3] recorded EEG signal streams from eighty healthy participants to investigate the association between task engagement and cognitive workload while performing learning and memory tasks. Another study first applied independent component analysis (ICA) to obtain the independent features from EEG, then predicted the cognitive workload based on these features [19]. So et al. [4] used short-term frontal EEG signals collected from twenty healthy volunteers performing four cognitive and motor tasks to evaluate the dynamic changes in mental workload. Although EEG signal can be assessed directly from the electrical activity of cortical and sub-cortical neurons with a very high temporal resolution in milliseconds, it is non-stationary and vulnerable to several types of noise such as blinking, frequency interference, and motion-related and sensor-based artifacts [20]. Several methods have been proposed to handle various types of noise appearing in EEG, including wavelet transform-based techniques, ICA-based methods, and adaptive filtering [21]. Usually, applying multiple denoising methods at the same time is necessary to handle multiple types of noise involved in EEG [21]. However, simultaneously applying several denoising algorithms not only increases the complexity of the denoising process but also leads to uncontrolled interactions between used algorithms [22]. In addition, the heavily used denoising algorithms potentially lead to the loss of relevant information in data. Therefore, denoising EEG signals usually requires practical experiences and sometimes needs manual processing steps [23].

### 2.2. HRV and BPV

There have been various works that leverage HRV parameters to predict cognitive workload. One study investigated real-time mental workload estimation via HRV parameters collected from 45 subjects [24]. Another study suggested that mental workload causes variations in non-linear HRV dynamics, which leads to increased risk of cardiac disorders [25]. Shakouri et al. [26] examined the correlation between HRV parameters and subjective workload during simulated driving in a NASA-TLX setup. There have been relatively few studies that link the cognitive workload with blood pressure variability (BPV). Stuiver et al. [27] investigated the effects of mental workload change in the BPV parameters collected from fifteen subjects during driving simulation. Hjortskov et al. [28] analyzed the impacts of mental stress with HRV and BPV and proposed that HRV is more sensitive to mental stress than BPV.

### 2.3. Eye Gaze

Eye gaze is another well-known indicator to assess human workload variations within a short time interval, making it a practical tool for predicting cognitive workload in real-time applications [29]. Considering that eye gaze is easier to collect in daily life situations and is less sensitive to undesired interference and motion artifacts than EEG, it is commonly used in workload estimation tasks [30,31]. Several studies used pupil diameter as a reliable indicator for assessing cognitive workload [30,32,33,34,35,36]. Pfleging et al. examined the correlation between pupil diameter and cognitive effort by adjusting the lighting conditions [33]. In another study, Palinko et al. suggested that eye tracking is one of the best approaches to predict cognitive workload in simulated driving, and pupil diameter is a reliable parameter to infer workload [30]. Bitkina et al. [35] investigated the efficiency of multiple eye tracking parameters including gaze fixation, pointing, and pupil diameter in workload estimation during simulated driving. Pang et al. [36] recorded several eye gaze parameters including fixation duration, blink duration, and pupil diameter to estimate different cognitive workload levels by adjusting the task difficulties. Additionally, some recent works evaluated eye gaze parameters for workload prediction in real-time applications [37,38]. Specifically, Kosch et al. [37] investigated real-time assessment of mental effort in the context of task complexity adaptation, while Appel et al. [38] used the normalized values of several eye gaze parameters such as pupil diameter, blink duration, and the number of blinks for online differentiation of cognitive workload.

### 2.4. Combination of Multiple Signal Modalities

There are also numerous works that aim to fuse multiple physiological signal types to boost the performance of mental workload estimation. For instance, the studies in [39,40,41] combined eye gaze and EEG signals to jointly predict the cognitive workload. Specifically, Khedher et al. [39] recorded both eye gaze and EEG signals from fifteen students in a virtual learning environment to classify two groups of learners: students who successfully resolved the tasks and students who did not. Additionally, their numerical results showed that the *k*-nearest neighbor classifier achieves the best classification efficiency over the other considered methods. Rozado and Dunser [40] combined the extracted features from EEG and pupil diameter to assess the cognitive workload [40]. Their results suggested that integrating EEG parameters with pupil diameter provides higher accuracy in workload classification compared to using a single signal type.

Even though combining multiple physiological signals may boost the accuracy of the trained model under some particular scenarios [40,42,43,44], it is not clear whether multi-modality learning, i.e., combining multiple physiological signals, can outperform single-modality learning in predicting the mental workload states. Indeed, some counter-examples appeared in the literature, practically pointing out that using multi-modality learning in workload prediction tasks may be harmful [41,45,46]. For example, Borys et al. [45] studied several combinations of EEG and pupillometry features to conclude that single model learning using eye-tracking data alone could achieve higher accuracy than other fusion-based models in cognitive workload classification tasks. Another study reviewed several research works on workload prediction from single modality and fusion of multiple signal modalities and concluded that most of the research works propose better performance in workload estimation by leveraging single modality compared to the fusion of multiple signal modalities [47]. They reported four studies within six that suggest that combining EEG and eye gaze does not enhance the overall performance in assessing workload over single usage of EEG or pupillometry, which is compatible with our findings.

### 2.5. Machine Learning for Detecting and Assessing Cognitive Workload

Machine learning models have been extensively used in literature for detecting and assessing cognitive workload. For instance, *k*-nearest neighbor was used in [39,48,49,50], naive Bayes was used in [51,52], random forest was used in [50,53], support-vector machines were used in [4,54,55,56,57,58], and neural network-based models were used in [49,50,54,59,60,61,62,63,64]. Since the performance of workload classification/prediction depends on learning models, previous works usually applied multiple learning models for detecting and assessing cognitive workload [39,65].

Finally, we refer the readers to recent surveys on using pupillometry, EEG, HRV, BPV, or their combinations for categorizing the mental workload in [16,47,66].

## 3. Experimental Data

We utilized pupillometry, EEG, and arterial blood pressure data from 80 subjects performing a multi-modal interactive driving task in a fixed-base driving simulator with an open-cab mockup (see [8,9] for details about the experimental setup). Ten braking events were generated, during which a vehicle appeared 200 m in front of the driver, causing the driver to brake quickly to avoid a collision. In addition, drivers had to answer 20 questions roughly every 30 to 60 s. Dialogue and braking events were arranged such that the time interval between the onset of the braking event and the end of the question, i.e., the “stimulus onset asynchrony” (SOA), was between −1 and +1 s with a step size of 0.5 s, for a total of five SOA conditions: SOA values of −1 and −0.5 represent cases where the braking event occurs 1 and 0.5 s after the completion of the question, respectively, while SOA values of 1 and 0.5 represent the cases where the braking event takes place 1 and 0.5 s before the completion of the question, respectively, and an SOA of 0 indicates that the braking event begins at the same time as the question ends.

### 3.1. Eye Gaze Recording

**Figure 1:**
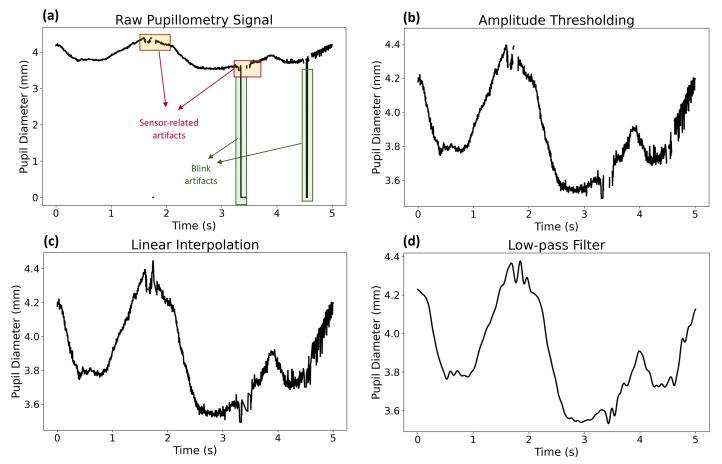
Pupillometry pre-processing steps: (**a**) raw pupil diameter signal, (**b**) signal after applying amplitude thresholding, (**c**) signal after applying linear interpolation, and (**d**) signal after applying Butterworth low-pass filter.

Eye gaze data was recorded with a Pupil Core (Pupil Labs, Berlin, Germany) eye tracker to obtain the pupillometry signal with a sampling rate of 400 Hz, which contains a 200 Hz binocular camera and a 120 Hz world camera. We performed three-step pre-processing to remove any out-of-band noise, sensory interference, and blink artifacts. First, we removed the signal segments with an amplitude of lower than 0.8 mm or greater than 10 mm by considering that the signal parts with an amplitude of lower than 0.8 mm are potential blink artifacts [67] and the quantitative pupil dilation widens up to 10 mm [68]. Second, we applied linear interpolation to fix the extracted parts [67]. Finally, we leveraged a fifth-order Butterworth low-pass filter with a cutoff frequency of 10 Hz to eliminate baseline wander [69]. Figure 1 illustrates the pre-processing steps of the pupillometry signal.

### 3.2. EEG Recording

EEG data was collected with an Enobio (Neuroelectrics, Cambridge, MA, USA) system with a 24-bit resolution and a sampling rate of 500 Hz with 3.14 cm${}^{2}$ silver/silver chloride electrodes located corresponding to the international 10-10 system (FC1, FC2, FC5, FC6, CP1, CP2, CP5, and CP6). To pre-process the EEG signals, we applied a three-stage denoising method. First, we applied a sixth-order Butterworth band-pass filter between 0.1 Hz and 30 Hz to eliminate the out-of-band noise [70,71]. Second, we used independent component analysis (ICA) to decompose the mixture of signal streams to its hidden components. ICA is implemented with the presumption that the latent components are non-Gaussian and statistically independent, and it is an effective tool for extracting the blink artifacts from EEG signals [72]. We identified the blink artifacts with the instantaneous spikes in the amplitude and manually removed the ICA components, which are correlated with blink artifacts. Third, we applied Kalman smoothing, which is a prominent method to predict the state of dynamic linear structures in the presence of noise [73]. To achieve this, we utilized the Python library “Tsmoothie” [74] to smooth the EEG signals.

### 3.3. Arterial Blood Pressure Recording

**Figure 2:**
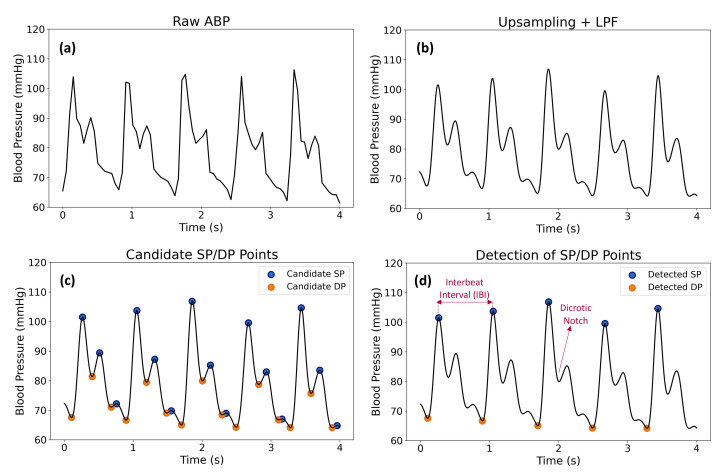
Arterial blood pressure (ABP) signal pre-processing and SP/DP point detection steps: (**a**) raw ABP signal, (**b**) signal after upsampling and applying law-pass filter, (**c**) the candidate SP/DP points, and (**d**) estimated SP/DP values.

A NIBP100D non-invasive fingertip plethysmography system was attached to participants’ left hands to collect the arterial blood pressure streams with a sampling rate of 20 Hz (BIOPAC Systems, Goleta, CA, USA). We pre-processed the arterial blood pressure (ABP) signal with three steps described in Figure 2. First, the ABP signal was upsampled to 250 Hz. Then, a Butterworth low-pass filter with a cutoff frequency of 5 Hz and an order of 5 was applied to eliminate the out-of-band noise. Finally, the systolic pressures (SP) and diastolic pressures (DP) were identified by applying both an amplitude threshold and a beat-to-beat duration threshold based on the behavior of cardiac dynamics. To achieve these, the candidates of SP and DP were obtained by taking the local maxima and local minima points from the ABP signal, respectively. The candidate SP points were evaluated as the potential heartbeats by considering that SP points taken from arterial blood pressure signals are reliable indicators to calculate heart rate and interbeat interval (IBI) [75,76,77]. Here, IBI represents the time interval between two consecutive heartbeats. Then, the amplitude threshold, which represents the threshold for the difference between the sequential DP and SP points, was determined as 10 mmHg. In other words, the subsequent DP and SP points whose amplitudes differ more than 10 mmHg were identified as possible SP and DP points. Next, minimum and maximum IBI values were determined to be 30 and 120 beats per minute (bpm), respectively. The intuition behind this selection was that the resting heart rate (HR) values of a normal adult remain within 60 bpm and 90 bpm [78]. We used a wider range of HR to eliminate the false detection of HR values based on subject-based variations.

## 4. Methods

### 4.1. Workload Evaluation

We generated three cognitive workload levels by combining the single driving event with additional tasks such as braking and dialogue: “Level 0” represents the baseline (i.e., the lowest level of cognitive workload while only driving without any additional task); “Level 1” represents the combination of the driving task and the dialogue task, where subjects had to answer questions like “What is your favorite color?”, “What pets do you have?”, “What sports do you watch?”, or “What type of movies do you like?”; and “Level 2” represent driving with simultaneous dialogue and braking events. To construct a reliable dataset for cognitive workload estimation/classification, we discarded the samples that were too noisy, leading to a balanced dataset of 348 samples, with each workload level containing 116 samples (from 43 healthy volunteers).

We used three SOA types within five to generate our dataset. Specifically, we did not include the SOA types of −1 and −0.5, which represent the case that the dialogue event ends before the onset of the braking event. The assumption behind this selection was that in the case of SOA events −1 and −0.5, the braking event starts after the completion of the question when the participant already thinks about the question and possibly generates the response in mind. Thus, the potential increase in cognitive workload based on the combination of braking and dialogue tasks would reduce. We, therefore, only leveraged the SOA types of 0, 0.5, and 1 to generate Level 2 in this study.

### 4.2. Pupillometry

We used the left pupillometry signal to obtain pupil dilation, the fixation count, and the fixation duration, assuming that the left and right pupil dilations are synchronous. We calculated percentage change in pupil size (PCPS) to avoid subject-based variations in pupil diameter by considering the range of pupil diameter fluctuations from person to person. The PCPS is calculated using the following equation [79]:

(1) $$\mathrm{PCPS}=\frac{\mathrm{Current}\;\mathrm{value}\;\mathrm{of}\;\mathrm{pupil}\;\mathrm{diameter}-\mathrm{Baseline}\;\mathrm{value}\;\mathrm{of}\;\mathrm{pupil}\;\mathrm{diameter}}{\mathrm{Baseline}\;\mathrm{value}\;\mathrm{of}\;\mathrm{pupil}\;\mathrm{diameter}}\times 100\%,$$

Here, we determined the baseline measure of the pupil diameter by calculating the average value of a 1 s signal segment before the onset of the stimulus. We took 2.5 s time frames from pre-processed pupillometry signals for each workload level to determine PCPS values. Next, we calculated the average PCPS (APCPS) as follows [79]:

(2) $$\mathrm{APCPS}=\frac{1}{M}\;\sum_{i=1}^{M}PCPS_{i},$$

where $PCPS_{i}$ represents the percentage change in pupil size for the *i*th sample, and *M* denotes the total number of samples in the time domain. In addition to PCPS and APCPS, we also obtained the number of fixations and calculated the mean of fixation duration within 2.5 s time windows.

### 4.3. Electroencephalography

To perform the statistical analysis of EEG, we calculated the power spectral density (PSD) of EEG streams to obtain the power distribution of the EEG signals in the frequency domain. To calculate the estimate of the PSD, we used Welch’s method, which partitions the data into segments to compute the estimation of the PSD by averaging the periodograms over segments [80,81]. To achieve this, we used non-overlapping 2 s windows with a sampling rate of 500 Hz. Then, we computed the absolute power corresponding to different sub-bands of EEG by defining the frequency ranges of each frequency band and approximating the area under the PSD curve. Based on that, we measured the average power of EEG signal from four frequency bands: 1 Hz to 4 Hz (δ), 4 Hz to 8 Hz (θ), 8 Hz to 12 Hz (α), and 12 Hz to 30 Hz (β), respectively. Here, based on the band-pass filter between 0.1 Hz and 30 Hz, which is suggested to eliminate the out-of-band noise from EEG signals [70,71] and given that within five frequency bands of EEG, δ, θ, and β are the most commonly used sub-bands for workload estimation [82,83,84], we excluded the gamma sub-band, which contains the frequencies higher than 30 Hz.

### 4.4. Arterial Blood Pressure

In this study, we considered the systolic pressures (SP) points as the heartbeats and used them to obtain the interbeat interval (IBI). We calculated the heart rate from the estimated IBI with the following equation:

(3) $$\mathrm{HR}=\frac{6\times 10^{4}}{\mathrm{IBI}\;\left(\mathrm{ms}\right)}\;\;\;(\mathrm{bpm}).$$

Based on the estimated HR and IBI values, we acquired two types of measurements: (1) heart rate variability (HRV) and (2) blood pressure variability (BPV) from 30 s time windows for each cognitive workload level.

#### 4.4.1. HRV

HRV is the variation in sequential IBIs over time. In this study, five time domain and six frequency domain HRV features were calculated from a 30 s ABP signal to investigate the performance of HRV in the assessment of cognitive workload.

**Time Domain HRV Features.** The time domain HRV features included the average of IBIs (meanIBI (ms)), the standard deviation of IBIs (SDIBI (ms)), the average of HR values (meanHR (bpm)), the standard deviation of HR values (SDHR (bpm)), and the root-mean-square of successive IBI differences (RMSSD). RMSSD was calculated with the following equation [85]:

(4) $$\mathrm{RMSSD}=\sqrt{\frac{1}{M-1}\sum_{t=1}^{M-1}{\left(IBI_{t+1}-IBI_{t}\right)}^{2}}\;\;(\mathrm{ms}).$$

where *M* and $IBI_{t}$ represent the number of heartbeats over time and the interbeat interval between time *t* and t+1, respectively.

**Frequency Domain HRV Features.** First, the cubic spline interpolation was used to interpolate the sequential IBI values. Then, the fast Fourier transform (FFT) was applied to obtain the sample frequencies and the frequency components were calculated from the one-side frequency range of FFT. Six frequency domain features were taken, which include very low frequency (VLF) power (in ${\mathrm{ms}}^{2}$), low frequency (LF) power (in ${\mathrm{ms}}^{2}$), high frequency (HF) power (in ${\mathrm{ms}}^{2}$), total power (in ${\mathrm{ms}}^{2}$), HF normalized unit (HFnu) (%), and LF normalized unit (LFnu) (%). VLF, LF, and HF power represent the power of the frequency components of IBI variation lower than 0.04 Hz, between 0.04–0.15 Hz, and between 0.15–0.4 Hz, respectively [86]. The total power is the sum of the powers from VLF, LF, and HF.

LFnu and HFnu were formally defined as follows:

(5) $$\mathrm{LFnu}=\frac{\mathrm{LF}}{\mathrm{LF}+\mathrm{HF}}\times 100(\%)$$

(6) $$\mathrm{HFnu}=\frac{\mathrm{HF}}{\mathrm{LF}+\mathrm{HF}}\times 100(\%)$$

#### 4.4.2. BPV

BPV is defined as the constant and dynamic variations in both systolic and diastolic blood pressures and was examined with different categories, such as long-term BPV (visit-to-visit), mid-term BPV (day-to-day), short-term BPV (over 24 h), and very short-term BPV (beat-to-beat) [87]. In this study, we took a 30 s ABP signal and calculated eight time domain BPV features together with twelve frequency domain BPV features to assess the very short-term BPV.

**Time Domain BPV Features.** We calculated four time domain BPV features, including the standard deviation (SD), successive variation (SV), coefficient of variation (CV), and average real variability (ARV). The definitions of time domain features were expressed with the following equations:

(7) $$\begin{array}{ccc} \mathrm{SD} & = & \sqrt{\frac{1}{N-1}\sum_{t=1}^{N}\left(x_{t}-\bar{x}\right)}\;\;(\mathrm{mmHg}), \end{array}$$

(8) $$\begin{array}{ccc} \mathrm{SV} & = & \sqrt{\frac{1}{N-1}\sum_{t=1}^{N-1}{\left(x_{t+1}-x_{t}\right)}^{2}}\;\;(\mathrm{mmHg}), \end{array}$$

(9) $$\begin{array}{ccc} \mathrm{CV} & = & \frac{\mathrm{SD}}{\bar{x}}\times 100(\%), \end{array}$$

(10) $$\begin{array}{ccc} \mathrm{ARV} & = & \frac{1}{N-1}\sum_{t=1}^{N}\left|x_{t+1}-x_{t}\right|\;\;(\mathrm{mmHg}), \end{array}$$

where *N*, $x_{t}$, and $\bar{x}$ represent the number of blood pressure values in time domain, the blood pressure value at time *t*, and the mean blood pressure, respectively [88].

**Frequency Domain BPV Features.** To determine the frequency domain BPV features, the cubic spline interpolation was applied to interpolate the SP and DP values. Next, the sample frequencies were determined via FFT, and a one-side frequency range of FFT was taken to calculate the frequency components. We calculated six frequency domain features of BPV, which contain very low frequency (VLF) power (in ${\mathrm{mmHg}}^{2}$), low frequency (LF) power (in ${\mathrm{mmHg}}^{2}$), high frequency (HF) power (in ${\mathrm{mmHg}}^{2}$), total power (in ${\mathrm{mmHg}}^{2}$), LFnu (%), and HFnu (%). Each feature was calculated for both SP and DP (12 features in total). VLF power, LF power, and HF power represent the power of the frequency components of blood pressure variation lower than 0.04 Hz, between 0.04–0.15 Hz, and between 0.15–0.4 Hz, respectively [89]. The total power is the sum of VLF, LF, and HF power [89]. LFnu and HFnu represent the LF and HF of blood pressure fluctuation in normalized units (both for SP and DP) [90], respectively. We used Equations (5) and (6) to calculate LFnu and HFnu for BPV.

### 4.5. Extracted Features of Pupillometry and EEG

#### 4.5.1. Power Spectral Density of EEG

One of the most widely used extracted features from EEG is power spectral density (PSD) [91,92,93,94]. PSD quantifies the amount of power distributed in each frequency band of EEG signal [95]. From the pre-processed EEG data, we extracted the PSD by measuring the power in four standard frequency bands θ, α, δ, and β, which was explained in detail in Section 4.3. Because each EEG sample has a length of 2.5 s, we used a periodogram with a non-overlapping rectangular window size of 2.5 s to estimate the average spectral power in our experiment. The average spectral power was then integrated over each EEG frequency band to generate the PSD data [93,94]. From eight EEG channels and four frequency bands, each PSD sample corresponded to a 32-dimensional vector.

#### 4.5.2. Extracted Feature for Pupillometry

Mean and variance have been considered as the most commonly extracted features for pupillometry signals in the literature [96,97,98]. Therefore, in this paper, we decided to compute the mean and variance of PCPS, which was taken from the pupillometry signal explained in Section 4.2, to generate its two-dimensional vector extracted features.

#### 4.5.3. Combinations of Extracted Features

To explore the effectiveness of the combination of multiple physiological signals in predicting workload levels, the extracted features of pupillometry and EEG were concatenated as the inputs for our learning models. Particularly, by concentrating the extracted features from pupillometry and EEG, each combined sample corresponds to a 34-dimensional vector. These combined signals will be used later in Section 6.2.2.

## 5. Machine Learning Methodologies

In this section, we employed different machine learning models to predict the cognitive workload under two distinct settings: (a) single-modality, i.e., using a single physiological signal as the input of learning models, and (b) multiple-modality, i.e., combining multiple physiological signals as the input of learning models. Since the models matter in assessing the classification performance on different types of data, we employed five machine learning models named *k*-nearest neighbor (*k*-NN), naive Bayes (NB), random forest (RF), support-vector machines (SVM), and a neural network-based model (NNM) to provide a better comparison. It is worth noting that these selected learning models have been widely used in literature for cognitive workload classification tasks. For instance, *k*-NN was used in [39,48,49,50], NB was used in [51,52], RF was used in [50,53], SVM was used in [4,54,55,56,57], and NNM was used in [49,50,59,60].

For model training and testing, we divided each dataset into two subsets, the training set and the test set, with the ratio of 80% and 20%, respectively. For hyper-parameter tuning, we utilized five-fold cross-validation [99] for all non-neural network-based models. Particularly, the training set was first split into five equally sized groups. Within these five groups, one group was alternatively selected as the validation set, while the rest of the four groups were considered as four sub-training sets. The model was trained on four sub-training sets and tested on the validation set five times until all groups were selected once as the validation set. The model that achieved the highest accuracy averaged over five validation sets was selected as the best model. After the determination of hyper-parameters from the best model, we trained from scratch using all training data and tested on the testing set to obtain the final classification accuracy. For the neural network-based model (NNM), we kept the setting the same except that we did not apply five-fold cross-validation. We performed model selection based on the validation accuracy produced by a fixed validation set and repeated the above procedure five times for all models with different random seeds. The details of our learning models are described below.

### 5.1. k-Nearest Neighbor

*k*-nearest neighbor is a supervised learning method that outputs the label of the tested sample based on the labels of its *k*-nearest neighbors, i.e., the *k* closest samples (from the training set) to the tested sample. In this paper, we consider the number of nearest neighbors *k* as a tunable hyper-parameter which will be scanned in the range of [1,30].

### 5.2. Naive Bayes

Naive Bayes is a supervised learning technique that relies on the well-known Bayes’ theorem together with a simple (naive) assumption that each input (sample) conditioned on its label is independent of each other. In the naive Bayes algorithm, the conditional distribution of the data given label can be modeled using five common probabilistic models named Gaussian naive Bayes, multinomial naive Bayes, complement naive Bayes, Bernoulli naive Bayes, and categorical naive Bayes. For example, in the Gaussian naive Bayes model, it is assumed that the conditional distribution of the data given label follows the Gaussian distribution. Because the conditional distribution of data and its label varies in practice, we consider the probabilistic models as tunable hyper-parameters.

### 5.3. Random Forest

Random forest is a supervised learning method that combines multiple decision trees trained from different samples and subsequently takes their majority vote to output to determine final estimation. The number of decision trees (or the number of predictors) used in the random forest method is considered a tunable hyper-parameter. Here, we select the number of decision trees in the range of [1,50].

### 5.4. Support-Vector Machine

A support-vector machine is a supervised learning method that maps training samples from the input space into the latent space to maximize the width of the gap between different categories (groups of samples having the same label). To deal with non-linear datasets, a kernel trick is usually applied to SVM to obtain an optimal projection such that the data is linearly separable in the projected space. Here, we considered the types of kernel function as tunable hyper-parameters and selected the best kernel among four common kernel functions named linear, polynomial, radial basis, and sigmoid.

### 5.5. Neural Network-Based Models (NNM)

Deep neural networks have been suggested to be efficient in extracting task-related characteristics and thus have been widely used in literature for cognitive workload classification [49,50,59,60,100,101,102]. Here, we mainly used multi-layer perceptron (MLP) neural networks to classify the cognitive workload for all types of signals. In addition, we utilize the recent developed time-series neural network models such as EEGNet [102] for EEG, multivariate long short-term memory fully convolutional network (MLSTM-FCN) [103] and InceptionTime [104] for both EEG and pupillometry. Details of MLP, EEGNet, MLSTM-FCN, and InceptionTime models are described below.

#### 5.5.1. Multi-Layer Perceptron

In this study, we used a multi-layer perceptron neural network composed of three fully connected layers followed by a linear layer with the output dimension of $T_{1}\to T_{2}\to T_{3}\to T_{4}\to n$, where $T_{1},T_{2},T_{3},T_{4}$ denote the size of the first, the second, the third fully connected layer, and the last linear layer, respectively, while *n* denotes the number of label classes, i.e., the number of workload levels that need to be classified. We used rectified linear unit (ReLU) [105] as the activation function. To stabilize the training process and avoid overfitting, we applied batch normalization and dropout in each fully connected layer. For EEG and pupillometry, we set $\left(T_{1},T_{2},T_{3},T_{4}\right)=\left(2048,1024,512,64\right)$ and $\left(T_{1},T_{2},T_{3},T_{4}\right)=\left(512,256,128,128\right)$, respectively. For other physiological parameters, such as fixation number, fixation duration, heart rate variability, and blood pressure variability, we used $\left(T_{1},T_{2},T_{3},T_{4}\right)=\left(32,32,16,16\right)$. The architecture of our MLP neural network can be found in Table 1.

#### 5.5.2. Additional Time-Series Learning Models

Motivated by the recently developed time-series neural network models in [102,103,104], we want to apply EEGNet [102] for EEG and use MLSTM-FCN [103] and InceptionTime [104] for both EEG and pupillometry.

**EEGNet.** In this paper, we adapted the architecture of EEGNet in [102] composed of three convolutional layers followed by a classifier to our EEG signal. Since EEGNet was specially designed for EEG only, we did not employ EEGNet for other physiological signals. As recommended in [102], the length of the temporal convolution of the first layer in EEGNet was set to 250, which is exactly half of the sampling rate of EEG (500 samples per second).

**MLSTM-FCN.** In this paper, we applied a multivariate long short-term memory fully convolutional network (MLSTM-FCN) to both EEG and pupillometry signals. The MLSTM-FCN model was built from two blocks: a long short-term memory (LSTM) block and a fully convolutional network (FCN) block [103]. The outputs from the FCN block and LSTM block were combined and fed into a linear classifier to produce the final classification output.

In our implementation, we kept all model parameters the same as the settings in [103]. However, to adapt to different types of signals, we changed the number of LSTM cells to eight for EEG and to one for pupillometry without applying a grid search.

**InceptionTime.** In this paper, we applied the InceptionTime learning model proposed in [104] to both EEG and pupillometry signals. The InceptionTime model was composed of five Inception networks. To stabilize the training model, each Inception network was initialized randomly [104]. In addition, the Inception network has integrated the ResNet modules [106] with the inception modules using different lengths of filters for diverse feature extraction [107]. The bottleneck layers [106] were further applied to reduce the model complexity and sped up the training process. In our implementation, we kept all the parameters of the model the same as the setting in [104].

It is worth noting that to stabilize the training process and avoid overfitting, batch normalization and dropout were employed for all neural network-based models including MLP, EEGNet, MLSTM-FCN, and InceptionTime. Finally, for hyper-parameter tuning, the Adam optimizer [108] was applied with the learning rate selected from $\left\{1\times 10^{-5},5\times 10^{-5},1\times 10^{-4},5\times 10^{-4}\right\}$. The batch size was picked from {32,64,128,256}. For all selected pairs of batch size and learning rate, the number of epochs was always set to 300.

## 6. Results and Discussion

To evaluate the effectiveness of different physiological signals in assessing the mental workload levels, we first performed a statistical analysis of the performance of various parameters obtained from different types of signal modalities, such as human gaze parameters (APCPS, fixation number, and fixation duration), EEG, HRV, and BPV. Next, the classification accuracies of several machine learning methodologies in three workload classification tasks (levels 0-1, 0-2, and 1-2) were reported to verify these statistical analyses.

### 6.1. Statistical Analysis of Physiological Signals

#### 6.1.1. Pupillometry

**Figure 3:**
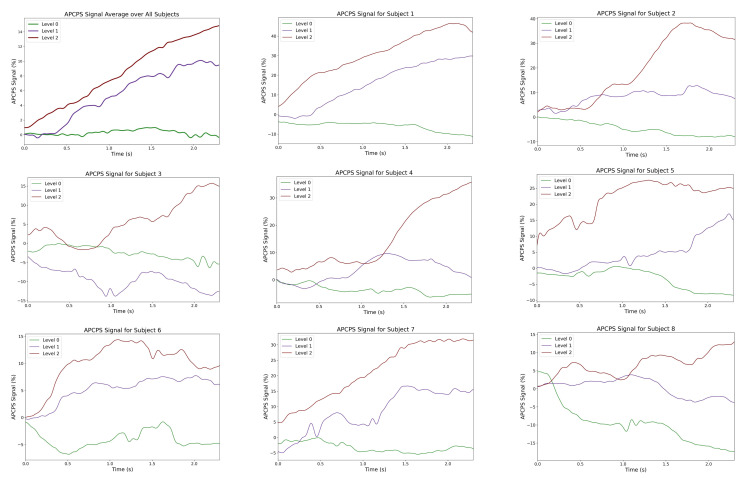
The variations in APCPS obtained from eight samples and the variation in mean APCPS over 116 samples (upper left) for three different workload levels.

We examined the variations in three human gaze parameters obtained from pupillometry signals, APCPS, mean fixation number, and mean fixation duration, for three workload levels. Figure 3 shows the change in the APCPS for eight samples obtained from different participants and the change in the mean APCPS over all samples within a 2.5 s time interval for various workload levels. The starting point represents the onset of baseline, dialogue, and SOA events for Level 0, Level 1, and Level 2, respectively. Our results indicate that a stable pattern is observed during Level 0, while the APCPS signal has distinctive patterns during Level 1 and Level 2 for multiple samples. Moreover, the mean APCPS over all samples remains around 0% for the baseline event, while it increases up around 10% and 15% for the dialogue and the SOA events, respectively. These results demonstrate the capability of the APCPS signal in differentiating different workload levels.

**Figure 4:**
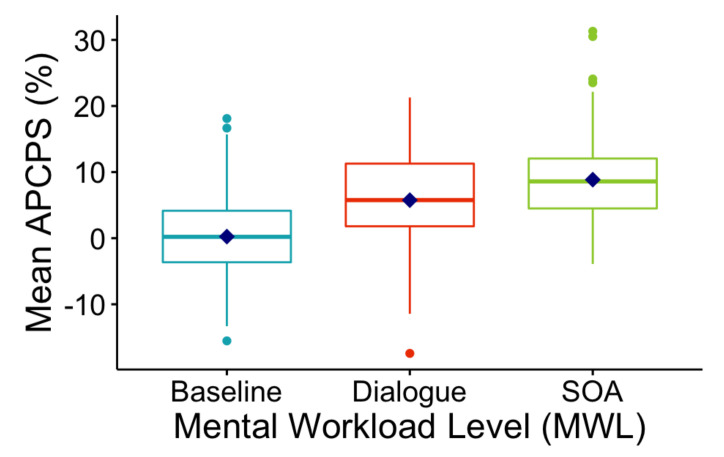
Boxplot of mean APCPS over all events for different cognitive workload levels.

Next, we calculated the mean APCPS, mean fixation number, and fixation duration over a 2.5 s time interval for each sample and investigated the outcomes to assess the change in these three human gaze parameters for various workload levels. To validate the direct correlation between mental effort and human gaze parameters, we performed a one-way ANOVA test for each human gaze parameter [109]. Table 2 illustrates the mean, median, standard deviation, and standard error of APCPS, average fixation number, and average fixation duration over 116 samples for three workload levels. Figure 4 also depicts the boxplot of the mean APCPS over 116 samples for three cognitive workload levels. The results demonstrate that the mean of the APCPS significantly increases with increased mental effort. The mean of the average fixation number is almost the same for Level 0 and Level 1, while it rises noticeably from Level 1 to Level 2. The mean of the average fixation duration decreases significantly from Level 0 to Level 1. However, there is a negligible difference between Level 1 and Level 2.

We further performed Tukey’s “honestly significant difference” (HSD) multiple pairwise comparison test [110] and the Benjamini–Hochberg test [111] to assess the differentiation between the three workload levels by leveraging three human gaze parameters. Table 3 illustrates *p*-values obtained from Tukey’s HSD and Benjamini–Hochberg tests for all pairs of workload levels at a significance level of 0.95. The results from both of the tests demonstrate that APCPS is the most efficient parameter within the three human gaze parameters to classify different pairs of workload levels. There is a statistically marked difference between all pairs of workload levels (≪0.05) in the case of using APCPS. On the other hand, the fixation number is capable of differentiating between Levels 0-2 and Levels 1-2; however, it looks like using the fixation number to distinguish between Levels 0-1 is practically impossible due to the small *p*-values of 0.33 and 0.14 obtained from both Tukey’s HSD and Benjamini–Hochberg tests, respectively. Moreover, although the fixation duration is efficient in classifying Levels 0-1 and 0-2 (*p*-values ≪0.05), it does not perform well in differentiating Levels 1-2. Based on the results, APCPS is the most efficient method of assessing the cognitive workload among these three morphological characteristics of pupillometry signals.

#### 6.1.2. Electroencephalography

To investigate the performance of EEG signal on workload prediction, we performed ANOVA, Tukey’s HSD multiple pairwise test, and the Benjamini–Hochberg test on four different frequency bands generated from PSD of EEG, δ, θ, α, and β. Table 4 illustrates the *p*-values of each pair of workloads for different frequency bands. The results demonstrate that within four EEG bands, α and β waves are more efficient in workload prediction and are capable of classifying only two pairs of workload levels, 0-1 and 0-2 (*p*-values < 0.05). While the θ wave is inefficient in discriminating any pairs of workload levels, the δ wave is able to differentiate the workload levels 1 and 2 with a *p*-value of $9\times 10^{-3}$ based on the Benjamini–Hochberg test. However, it is worth noting that none of the frequency bands is competent for the classification of all pairs of workload levels.

#### 6.1.3. Heart Rate Variability

**Figure 5:**
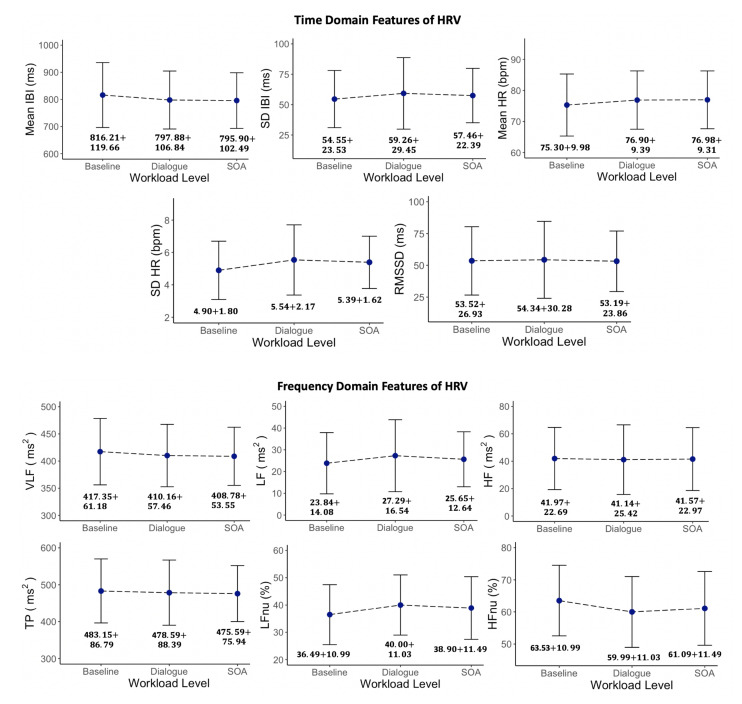
Analysis of HRV time domain and frequency domain features for different workload levels.

We performed statistical analysis on five time domain and six frequency domain HRV features to explore the efficiency of HRV features on workload classification. Figure 5 shows the fluctuations of HRV features in the presence of different workload levels. The HRV results indicate that only one time domain feature (SD HR) and two frequency domain features (LFnu and HFnu) have remarkable changes from Level 0 to Level 1. On the other hand, none of the HRV characteristics are capable of discriminating between Level 1 and Level 2. In addition, we run ANOVA Tukey’s HSD multiple pairwise test (with a confidence level of 0.95) on each HRV feature for every pair of workload levels to explore the efficiency of HRV features in classifying different pairs of workload levels. The *p*-values obtained from Tukey’s HSD multiple pairwise test for levels 0-1, 0-2, and 1-2 can be viewed in Table 5. The results demonstrate that only three HRV parameters (SD HR, LFnu, and HFnu) have *p*-values lower than 0.05 and can differentiate between Level 0 and Level 1, which is compatible with the results presented in Figure 5.

#### 6.1.4. Blood Pressure Variability

**Figure 6:**
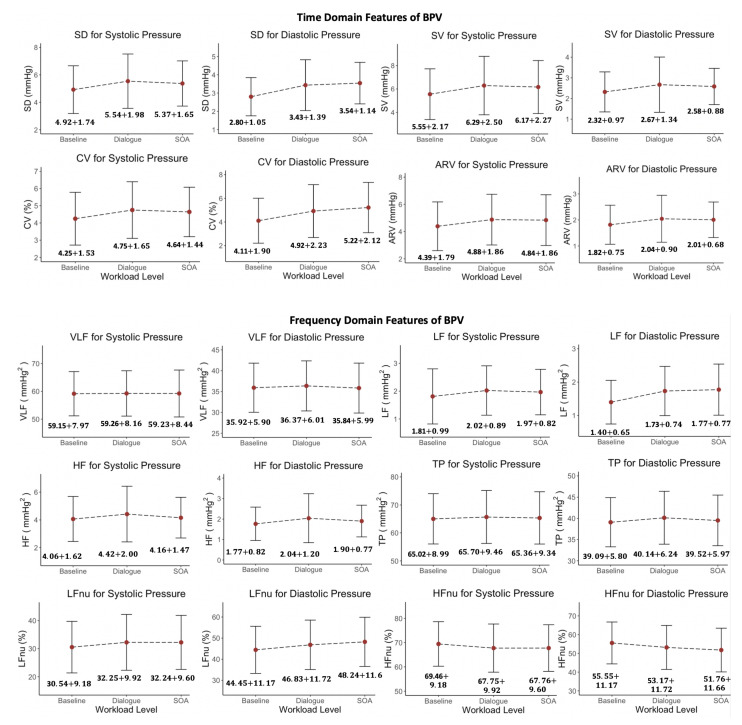
Analysis of BPV time domain and frequency domain features for different workload levels.

We also ran a statistical analysis on blood pressure variability (BPV) parameters to investigate the performance of time domain and frequency domain features of the BPV signal in assessing the cognitive workload levels. Figure 6 illustrates the variations of time domain and frequency domain BPV features with different workload levels. The variations of BPV in time domain indicate that only SD and CV for diastolic pressure can separate the three workload levels, while the rest of the time domain BPV features cannot distinguish between Level 0, Level 1, and Level 2. The variations of BPV in the frequency domain show that LF and LFnu for diastolic pressure increase with increased workload levels, and HFnu for diastolic pressure decreases with increased workload levels. The rest of the frequency domain BPV features do not have compatible patterns with workload degree alteration. Moreover, we also ran ANOVA Tukey’s HSD multiple pairwise tests (with a confidence level of 0.95) on each BPV feature for every pair of workload levels. Table 6 indicates the *p*-values for workload level pairs of 0-1, 0-2, and 1-2 of 20 different BPV features. The results show that among 20 BPV features, only four features (SD DP, CV DP, and LF DP) can differentiate Levels 0-1 and 0-2 (*p*-value <0.05). Furthermore, we observed that the features obtained from diastolic powers from arterial blood pressure signals are more efficient in classifying between different workload level pairs. There are only three BPV features acquired from systolic power that can differentiate Levels 0-1, which are SD SP (*p*-value =0.02), SV SP (*p*-value =0.04), and CV SP (*p*-value =0.03). Furthermore, any of the BPV features are able to distinguish between Level 1 and Level 2.

### 6.2. Classification Performance

As previously discussed in Section 5, in this section, we employed five different machine learning models to evaluate the performance of mental workload classification under (a) single-modality settings and (b) multi-modality settings. To shorten the notations, we used PCPS to denote the percentage change in pupil size of pupil diameter signal, BPV to denote blood pressure variability parameters, HRV to denote heart rate variability parameters, and FIX to denote fixation parameters including fixation number and fixation duration.

#### 6.2.1. Single-Modality Learning

Under the single-modality learning framework, we performed three classification tasks: (1) the 0-1 task, i.e., differentiating between workload level 0 and workload level 1, (2) the 0-2 task, i.e., distinguishing between workload level 0 and workload level 2, and (3) the 1-2 task, i.e., differentiating between workload level 1 and workload level 2.

Five learning methods, *k*-NN, NB, RF, SVM, and MLP, were applied for all types of signals, while MLSTM-FCN and InceptionTime were used for EEG and pupil diameter, and EEGNet was used for EEG only. All the experiments were repeated five times, and only the average accuracy together with its standard deviation will be reported.

The performance of the *k*-NN, NB, RF, SVM, and MLP models in task 0-1, task 0-2, and task 1-2 are shown in Table 7, Table 8, and Table 9, respectively. As seen, the highest accuracy in the 0-1 task is 74.47%, which was achieved by using PCPS with RF. The highest accuracy in the 0-2 task is 80.45%, which was achieved by using PCSP with SVM, while the highest accuracy in the 1-2 task is only 67.52%, which was achieved by using PCSP with MLP. The low accuracy in the 1-2 task can be explained by the way workloads level 1 and level 2 were constructed. Indeed, by the construction, level 2 was generated from level 1 by adding braking events that occurred frequently and did not cause a significant increase in cognitive workload in a short period of time (please see Table 1). Therefore, there was not much difference between the amount of workload contained in level 1 and level 2, leading to the low classification accuracy of task 1-2. Over three tasks, one can observe that PCPS outperformed other physiological signals regardless of learning models.

The performances of other learning models (MLSTM-FCN, InceptionTime, and EEGNet) when applied to EEG and pupil diameter signals are shown in Table 10, Table 11, and Table 12, respectively. Note that we used EEGNet for the EEG signal only but applied MLSTM-FCN and InceptionTime for both EEG and pupil diameter. Again, the PCPS can achieve a higher classification accuracy over all three workload classification tasks regardless of the learning models.

In conclusion, the observed results from Table 7, Table 8, Table 9, Table 10, Table 11 and Table 12 agreed with our previous analysis in Section 6.1, demonstrating that the eye gaze, particularly, the PCPS, is a sufficient modality for assessing human cognitive workload in interactive, multi-modal, multi-task settings.

#### 6.2.2. Multi-Modality Learning

For multi-modality learning, we decided to utilize two common approaches named data-level fusion and feature-level fusion to jointly learn from multiple physiological signals. The data-level fusion approach aims to combine two time-series signals together as the input of the learning models. Specifically, we separately used two MLP networks to learn the useful features from two physiological signals; then the outputs of these two networks were fed into the third MLP network to output the final labels. On the other hand, in the feature-level fusion approach, we simply concentrated the handcrafted extracted features from two different signals as the input for our learning models. Particularly, we used PSD as the extracted features for EEG and used mean and variance as the extracted features for PCPS. The details of the feature extraction process can be found in Section 4. Due to EEG and pupil diameter (PCPS) being the two most common physiological indicators for assessing the cognitive workload which have demonstrated their efficiency in literature, we focused on integrating these two signals together.

The performances of multi-modality learning for three workload classification tasks, 0-1, 0-2, and 1-2, can be viewed from Table 13. As seen, combining EEG with PCPS provided an improvement over using EEG alone; however, this combination did not show any improvement compared to only using PCPS. Particularly, the highest accuracies for EEG over three classification tasks. 0-1, 0-2, and 1-2, were 62.92∓8.64, 63.75∓5.63, and 61.76∓1.84, which were increased to 65.50∓0.83, 77.08∓3.22, and 62.90∓2.85 after being combined with pupil-diameter. In contrast, the highest accuracies of using pupil diameter alone over three classification tasks, 0-1, 0-2, and 1-2, were 74.47∓3.01, 80.45∓3.15, and 67.52∓6.35, which were substantially higher than the highest accuracy of using multi-modality learning, at 65.50∓0.83, 77.08∓3.22, and 62.90∓2.85, respectively.

The numerical results from Table 7, Table 8, Table 9, Table 10, Table 11, Table 12 and Table 13 supported our conclusion that combining multiple physiological signals did not significantly improve the quality of workload prediction over just using pupil diameter alone. It is worth noting that our conclusion agrees with the observations in [41,45,46].

For the convenience of the reader, our numerical results were summarized in Table 14, which indicates the highest accuracy of the tested signals (PCPS, EEG, FIX, BPV, HRV, and PCPS+EEG) and its standard deviation over three classification tasks, 0-1, 0-2, and 1-2, respectively.

Finally, the neural network-based models (multi-layer perceptron, EEGNet [102], multivariate long short-term memory fully convolutional network (MLSTM-FCN) [103] and InceptionTime [104]) required approximately 20 min to finish the whole training process, while the non-neural network-based models (k-nearest neighbor, naive Bayes, random forest, and support-vector machine) usually required around one minute. Note that our dataset was small with just 348 samples and we ran all models on a Macbook pro 2.3 GHz, Quad-Core Intel Core i7, 32 GB 3733 MHz.

## 7. Conclusions

The goal of this paper was to investigate the utility of multiple physiological signals such as EEG, pupillometry, and arterial blood pressure, alone or combined, for assessing human cognitive workload in multi-modal, multi-task human-robot interaction contexts. Our statistical analyses and modeling results showed that (1) pupil dilation is the most effective physiological signal for assessing different cognitive workload levels and that (2) no fusion model combining multiple features could improve the prediction accuracy over pupillometry alone in three workload classification tasks. Since eye gaze data is practically easy to obtain and process compared to other physiological signals such as EEG and arterial blood pressure, our findings suggested that eye gaze could be the modality of choice for interactive assistive robots to infer human workload and use it to improve human-robot interactions.

## 8. Limitations and Future Work

Our study has some limitations. First, the current design only provided an offline determination of cognitive workload. Future works will evaluate the utility of real-time assessments of human workload to adapt robot behavior in human-robot interaction contexts. Second, our study only focused on examining the performance of different sensor modalities in predicting cognitive workload. However, we believe that other cognitive states, such as a sense of urgency, mind wandering, and interference, could be detected in the dataset as well. Finally, our results were limited by the subjects used in the experiment who had an average age of 20 and were recruited mostly from undergraduate/graduate programs at Tufts University. Hence, while we believe that the results will generalize to other populations (e.g., as the cognitive load would only be exacerbated in older populations), additional experiments need to be performed to verify this conjecture.

## Figures and Tables

**Table 1:** Architecture of the used multi-layer perceptron. *N* is the number of samples in one batch, $T_{1},T_{2},T_{3},T_{4}$ denote sizes of four layers, and *n* is the number of outputted classes.

Layer	Operation	Output Size
Input	-	(*N*, $T_{1}$)
The first FC layer	Linear($T_{1}$, $T_{2}$) + BatchNorm + ReLU + Dropout(0.25)	(*N*, $T_{2}$)
The second FC layer	Linear($T_{2}$, $T_{3}$) + BatchNorm + ReLU + Dropout(0.25)	(*N*, $T_{3}$)
The third FC layer	Linear($T_{3}$, $T_{4}$) + BatchNorm + ReLU + Dropout(0.25)	(*N*, $T_{4}$)
The last linear layer	Linear($T_{4}$, *n*)	(*N*, *n*)

**Table 2:** Statistical results of mean, median, standard deviation, and standard error for APCPS, fixation number, and fixation duration for different workload levels over all events.

	Workload Level	Mean	Median	Std	SE
APCPS	Level 0	0.24	0.21	6.08	0.56
	Level 1	5.75	5.76	7.36	0.68
	Level 2	8.85	8.59	6.71	0.62
Fixation Number	Level 0	7.30	7.00	3.32	0.31
	Level 1	7.99	8.00	3.70	0.34
	Level 2	10.10	10.00	4.05	0.38
Fixation Duration	Level 0	0.28	0.21	0.31	0.03
	Level 1	0.19	0.13	0.16	0.01
	Level 2	0.18	0.13	0.22	0.02

**Table 3:** Results of Tukey’s HSD multiple pairwise test and Benjamini–Hochberg (B & H) test for APCPS, fixation number, and fixation duration for different pairs of cognitive workload levels.

	Workload Level	*p*-Value (Tukey’s HSD)	*p*-Value (B–H)
APCPS	Level 0-Level 1	<1 × $10^{-7}$	3.6 × $10^{-9}$
	Level 0-Level 2	<1 × $10^{-7}$	2.0 × $10^{-16}$
	Level 1-Level 2	1 × $10^{-3}$	9.3 × $10^{-4}$
Fixation Number	Level 0-Level 1	0.33	0.14
	Level 0-Level 2	5 × $10^{-8}$	7.5 × $10^{-8}$
	Level 1-Level 2	5 × $10^{-5}$	6.6 × $10^{-5}$
Fixation Duration	Level 0-Level 1	7 × $10^{-3}$	6 × $10^{-3}$
	Level 0-Level 2	3 × $10^{-3}$	6 × $10^{-3}$
	Level 1-Level 2	0.96	0.76

**Table 4:** *p*-values from Tukey’s HSD multiple pairwise test and Benjamini–Hochberg test performed on four frequency bands obtained from PSD of EEG for different pairs of cognitive workload levels.

	Workload Level	δ	θ	α	β
Tukey’s HSD	Level 0-Level 1	0.06	0.79	0.02	$4\times 10^{-5}$
	Level 0-Level 2	0.88	0.71	$1\times 10^{-3}$	$3\times 10^{-5}$
	Level 1-Level 2	0.16	0.98	0.67	0.99
Benjamini–Hochberg	Level 0-Level 1	0.05	0.84	0.03	$3\times 10^{-4}$
	Level 0-Level 2	0.69	0.84	$9\times 10^{-3}$	$3\times 10^{-4}$
	Level 1-Level 2	$9\times 10^{-3}$	0.86	0.16	0.93

**Table 5:** *p*-values from Tukey’s HSD multiple pairwise test for different pairs of workload levels obtained from HRV parameters.

HRV Features	Level 0-Level 1	Level 0-Level 2	Level 1-Level 2
Mean IBI (ms)	0.41	0.34	0.99
SD IBI (ms)	0.33	0.66	0.85
Mean HR (bpm)	0.41	0.38	0.99
SD HR (bpm)	0.02	0.11	0.82
RMSSD (ms)	0.97	0.99	0.94
VLF (${\mathrm{ms}}^{2}$)	0.61	0.49	0.98
LF (${\mathrm{ms}}^{2}$)	0.16	0.61	0.66
HF (${\mathrm{ms}}^{2}$)	0.96	0.99	0.99
TP (${\mathrm{ms}}^{2}$)	0.91	0.79	0.97
LFnu (%)	0.04	0.22	0.73
HFnu (%)	0.04	0.22	0.73

**Table 6:** *p*-values from Tukey’s HSD multiple pairwise test for different pairs of workload levels obtained from BPV parameters.

BPV Features	Level 0-Level 1	Level 0-Level 2	Level 1-Level 2
SD SP (mmHg)	0.02	0.14	0.75
SD DP (mmHg)	$2.1\times 10^{-4}$	$1\times 10^{-5}$	0.76
SV SP (mmHg)	0.04	0.11	0.91
SV DP (mmHg)	0.04	0.15	0.81
CV SP (%)	0.03	0.13	0.85
CV DP (%)	$8.8\times 10^{-3}$	$1.8\times 10^{-4}$	0.53
ARV SP (mmHg)	0.11	0.15	0.99
ARV DP (mmHg)	0.06	0.15	0.93
VLF SP (${\mathrm{mmHg}}^{2}$)	0.99	0.99	0.99
VLF DP (${\mathrm{mmHg}}^{2}$)	0.84	0.99	0.78
LF SP (${\mathrm{mmHg}}^{2}$)	0.17	0.39	0.88
LF DP (${\mathrm{mmHg}}^{2}$)	$1.3\times 10^{-3}$	$2.5\times 10^{-4}$	0.90
HF SP (${\mathrm{mmHg}}^{2}$)	0.26	0.91	0.48
HF DP (${\mathrm{mmHg}}^{2}$)	0.07	0.53	0.50
TP SP (${\mathrm{mmHg}}^{2}$)	0.84	0.96	0.96
TP DP (${\mathrm{mmHg}}^{2}$)	0.38	0.85	0.71
LFnu SP (%)	0.37	0.37	0.99
LFnu DP (%)	0.26	0.03	0.62
HFnu SP (%)	0.37	0.37	0.99
HFnu DP (%)	0.26	0.03	0.62

**Table 7:** Classification accuracy of task 0-1 using *k*-NN, NB, RF, SVM, and MLP.

Signals	*k*-NN	NB	RF	SVM	MLP
PCPS	67.02 ∓ 4.88	73.40 ∓ 5.53	**74.47** ∓ 3.01	72.34 ∓ 2.61	67.92 ∓ 6.84
BPV	53.19 ∓ 3.98	59.57 ∓ 5.42	57.45 ∓ 7.82	55.32 ∓ 6.89	60.42 ∓ 6.42
HRV	50.53 ∓ 2.32	55.85 ∓ 4.35	55.85 ∓ 5.50	55.85 ∓ 2.56	55.00 ∓ 6.35
Fix	52.13 ∓ 1.06	54.79 ∓ 2.32	53.19 ∓ 2.61	57.98 ∓ 2.32	61.67 ∓ 4.80
EEG	54.19 ∓ 4.26	52.13 ∓ 3.19	60.11 ∓ 3.80	60.11 ∓ 9.07	62.92 ∓ 8.64

**Table 8:** Classification accuracy of task 0-2 using *k*-NN, NB, RF, SVM, and MLP.

Signals	*k*-NN	NB	RF	SVM	MLP
PCPS	79.26 ∓ 6.45	79.78 ∓ 7.44	70.68 ∓ 8.43	**80.45** ∓ 3.15	74.58 ∓ 3.42
BPV	59.57 ∓ 3.80	58.51 ∓ 4.39	58.51 ∓ 4.39	63.30 ∓ 6.45	63.75 ∓ 3.16
HRV	51.60 ∓ 3.80	56.38 ∓ 8.03	48.94 ∓ 3.98	54.72 ∓ 1.76	54.58 ∓ 3.73
Fix	61.17 ∓ 3.80	64.36 ∓ 9.32	58.51 ∓ 2.38	66.49 ∓ 5.07	63.33 ∓ 6.18
EEG	60.64 ∓ 1.84	54.54 ∓ 6.45	62.77 ∓ 7.74	57.98 ∓ 6.27	63.75 ∓ 5.63

**Table 9:** Classification accuracy of task 1-2 using *k*-NN, NB, RF, SVM, and MLP.

Signals	*k*-NN	NB	RF	SVM	MLP
PCPS	59.57 ∓ 4.63	60.63 ∓ 3.19	66.49 ∓ 10.02	63.11 ∓ 2.31	**67.52** ∓ 6.35
BPV	43.09 ∓ 2.76	44.68 ∓ 8.38	53.19 ∓ 3.36	48.81 ∓ 5.42	49.17 ∓ 1.14
HRV	47.87 ∓ 6.29	53.19 ∓ 7.37	49.47 ∓ 1.76	45.74 ∓ 6.29	49.58 ∓ 3.42
Fix	50.32 ∓ 3.36	53.85 ∓ 3.15	50.00 ∓ 6.81	58.12 ∓ 3.77	56.25 ∓ 6.07
EEG	57.98 ∓ 2.32	53.19 ∓ 5.83	59.77 ∓ 3.19	61.76 ∓ 1.84	61.33 ∓ 6.77

**Table 10:** Classification accuracy of task 0-1 using MLSTM-FCN, InceptionTime, and EEGNet.

Signals	MLSTM-FCN	InceptionTime	EEGNet
EEG	54.16 ∓ 7.80	54.58 ∓ 2.72	54.07 ∓ 4.03
PCPS	64.58 ∓ 2.95	67.50 ∓ 3.16	-

**Table 11:** Classification accuracy of task 0-2 using MLSTM-FCN, InceptionTime, and EEGNet.

Signals	MLSTM-FCN	InceptionTime	EEGNet
EEG	55.00 ∓ 5.23	53.33 ∓ 2.80	53.33 ∓ 6.69
PCPS	73.28 ∓ 5.24	57.98 ∓ 9.13	-

**Table 12:** Classification accuracy of task 1-2 using MLSTM-FCN, InceptionTime, and EEGNet.

Signals	MLSTM-FCN	InceptionTime	EEGNet
EEG	54.22 ∓ 8.70	53.91 ∓ 4.19	52.70 ∓ 3.27
PCPS	63.38 ∓ 4.75	56.25 ∓ 9.51	-

**Table 13:** Classification accuracy of tasks 0-1, 0-2, and 1-2 using data-level fusion and feature-level fusion approaches.

Signals	Tasks	Data-Level	Feature-Level
PCPS + EEG	0-1	65.50 ∓ 0.83	65.25 ∓ 6.52
PCPS + EEG	0-2	77.08 ∓ 3.22	66.51 ∓ 6.46
PCPS + EEG	1-2	62.90 ∓ 2.85	60.57 ∓ 8.52

**Table 14:** The highest accuracy and its standard deviation of tested signals (PCPS, EEG, FIX, BPV, HRV, and PCPS+EEG) over three classification tasks 0-1, 0-2, and 1-2, respectively.

Signals	0-1	0-2	1-2
PCPS	74.47 ∓ 3.01	80.45 ∓ 3.15	67.52 ∓ 6.35
EEG	62.92 ∓ 8.64	63.75 ∓ 5.63	61.76 ∓ 1.84
FIX	61.67 ∓ 4.80	66.49 ∓ 5.07	58.12 ∓ 3.77
BPV	60.42 ∓ 6.42	63.75 ∓ 3.16	53.19 ∓ 3.36
HRV	55.85 ∓ 4.35	54.58 ∓ 3.73	56.38 ∓ 8.03
PCPS+EEG	65.55 ∓ 0.83	77.08 ∓ 3.22	62.90 ∓ 2.85

## Data Availability

Not applicable.
